# ER stress and hepatic lipid metabolism

**DOI:** 10.3389/fgene.2014.00112

**Published:** 2014-05-09

**Authors:** Huiping Zhou, Runping Liu

**Affiliations:** ^1^Department of Microbiology and Immunology, School of Medicine, Virginia Commonwealth University, RichmondVA, USA; ^2^McGuire Veterans Affairs Medical Center, RichmondVA, USA

**Keywords:** ER stress, UPR, hepatic, lipid metabolism, liver diseases

## Abstract

The endoplasmic reticulum (ER) is an important player in regulating protein synthesis and lipid metabolism. Perturbation of ER homeostasis, referred as “ER stress,” has been linked to numerous pathological conditions, such as inflammation, cardiovascular diseases, and metabolic disorders. The liver plays a central role in regulating nutrient and lipid metabolism. Accumulating evidence implicates that ER stress disrupts lipid metabolism and induces hepatic lipotoxicity. Here, we review the major ER stress signaling pathways, how ER stress contributes to the dysregulation of hepatic lipid metabolism, and the potential causative mechanisms of ER stress in hepatic lipotoxicity. Understanding the role of ER stress in hepatic metabolism may lead to the identification of new therapeutic targets for metabolic diseases.

## INTRODUCTION

Endoplasmic reticulum is an important intracellular organelle responsible for protein synthesis, folding, modification, and trafficking. In addition, the ER also plays a crucial role in calcium homeostasis and in regulating the biosynthesis of steroids, lipids and carbohydrates (Borgese et al., 2006). In the ER, millions of proteins are synthesized, but not all of them are able to be properly folded and processed. Under normal physiological conditions, the unfolded or misfolded proteins are directed to degradation pathways through activating an evolutionally conserved signaling pathway, the UPR. Activation of the UPR can either help eliminate the unfolded proteins and restore cellular homeostasis, or activate a cascade of intracellular events resulting in cell death (Shen et al., 2004; Merksamer and Papa, 2010). The UPR is of particular importance in hepatocytes, which are rich in ER content and responsible for the synthesis of proteins, cholesterol, bile acids, and phospholipids. The UPR and its contribution to hepatic injury have been investigated in various liver diseases including ALD, NAFLD, DILD, cholestatic liver disease, and viral hepatitis (Zhou et al., 2006; Kaplowitz et al., 2007; Colgan et al., 2011; Dara et al., 2011; Jo et al., 2013).

## ER STRESS AND THE UPR

The ER is a membranous network of cisternae responsible for the synthesis and export of proteins and lipids. It is also crucial for cellular calcium homeostasis (Borgese et al., 2006; Gorlach et al., 2006). The ability of the ER to adapt to the metabolic changes, such as an increase in protein synthesis and accumulation of unfolded proteins and cholesterol in the ER lumen, is of paramount importance for the cell. When the misfolded or unfolded proteins accumulate in the ER, the ER stress and its related signaling pathways, UPR, are activated (Ron and Walter, 2007; Schroder, 2008).

**FIGURE 1:**
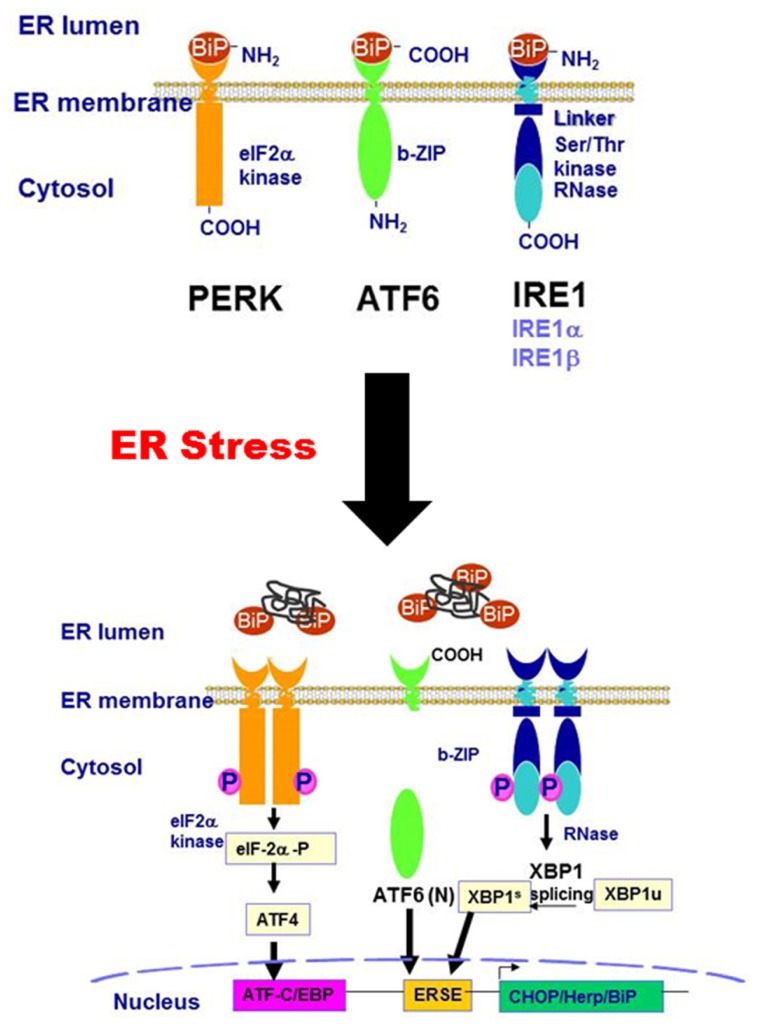
**Schematic diagram of the ER stress-activated signaling pathways**.

Three main branches of the UPR-mediated signaling pathways have been identified so far: the IRE1 pathway, protein kinase RNA-like ER kinase (PERK) pathway, and ATF6 pathway. As illustrated in **Figure 1**, IRE1, PERK, and ATF6 are associated with the ER membrane. Under non-stressed condition, these transmembrane proteins are bound to a chaperone protein, BiP/GRP78, which is also known as the master regulator of the UPR. The binding of BiP/GRP78 to these UPR transducers prevents them from activation. When the ER is stressed by accumulation of misfolded or unfolded proteins, depletion of ER calcium content, or increase of free cholesterol in the ER lumen, BiP/GRP78 is released from the UPR transducers. The disassociation of BiP/GRP78 from the UPR transducers results in the activation of IRE1-, PERK-, and ATF6-mediated signaling pathways. PERK activation results in a rapid down-regulation of protein synthesis *via* phosphorylation of eIF-2α and inhibition of the formation of the translation initiation complex (DuRose et al., 2009). The phosphorylated eIF-2α further promotes the translation of ATF4, a member of the basic leucine zipper (bZIP)-containing protein subfamily. IRE1α has both protein kinase and endoribonuclease activities. Under ER stress conditions, IRE1α is oligomerized and autophosphorylated. The activated IRE1α removes a 26-bp intron from the XBP1 mRNA, resulting in the production of spliced XBP1 protein (XBP1s). XBP1s, which is also a bZIP transcription factor, regulates the expression of several genes involved in UPR and ER-assisted degradation (ERAD) to help restore ER homeostasis (Acosta-Alvear et al., 2007). In addition, IRE1α also induces the activation of stress kinases, JNK and p38 MAPK, that promote apoptosis (Ron and Hubbard, 2008). ATF6 is the third branch of the UPR. Dissociation of BiP/GRP78 from ATF6 leads to its translocation to the Golgi, where it is processed into its active form by cleavage of its N-terminal domain by S1P and S2P (Chen et al., 2002). The activated ATF6 (ATF-6 N terminal domain) is translocated to the nucleus and functions as a transcription factor, promoting the expression of downstream target genes involved in ER stress including XBP1, GADD153 (also known as CHOP), and ER chaperones (Oyadomari and Mori, 2004). CHOP is a proapoptotic transcription factor that plays a critical role in ER stress-mediated apoptosis (Marciniak et al., 2004).

## HEPATIC LIPID METABOLISM

Liver is the central metabolic organ and plays a critical role in fatty acid and cholesterol metabolism (Hylemon et al., 2001). Several inter-dependent pathways are involved in hepatic lipid metabolism. The hepatic fatty acids can be derived from *de novo* lipogenesis, hydrolysis of triglyceride from cytoplasmic lipid droplets or direct uptake of non-esterified fatty acids from circulation (Bechmann et al., 2012). The liver is also a major organ in the processing of lipids into the various lipoproteins, in particular VLDL and LDL. Fatty acids synthesized by the liver are converted into triglyceride and exported as constituents of VLDL into the blood circulation. The VLDLs absorbed into peripheral tissues are partially digested by lipoprotein lipase into LDL and free fatty acids. LDL is transported into the cell *via* LDL receptors for its conversion into free fatty acids, cholesterol, and other components of LDL. Similarly, cholesterol also can be derived from *de novo* synthesis or absorbed from the diet, and are transported into circulation as lipoprotein particles (Horton et al., 2002a; Babin and Gibbons, 2009; van Heyningen, 2009; Musso et al., 2013; Faust and Kovacs, 2014). The cholesterol can be stored in cells as cholesterol esters or metabolized into bile acids. The hepatic triglycerides and cholesterol contents are tightly regulated by multiple interrelated signaling pathways. Under normal physiological conditions, lipid input is equal to lipid output from the body. Disruption of either the input or output pathways will result in dysregulation of lipid metabolism (Hylemon et al., 2001). Tremendous studies have been done to elucidate the extremely complex regulation network of hepatic lipid homeostasis (Bradbury and Berk, 2004; Bradbury, 2006; Weickert and Pfeiffer, 2006; Nguyen et al., 2008; Musso et al., 2009; Sparks and Dong, 2009; van Heyningen, 2009; Trauner et al., 2010; Jump, 2011; Fu et al., 2012). Here, we will focus on the current understanding regarding the role of ER stress in hepatic lipid metabolism.

## UPR AND HEPATIC LIPID HOMEOSTASIS

The ER is the primary site of lipid metabolism. Many enzymes and regulatory proteins of lipid metabolism reside in the ER. Perturbation of ER homeostasis contributes to hepatic steatosis, inflammation and insulin resistance in the liver (Kaplowitz et al., 2007; Hotamisligil, 2010; Rohrl et al., 2014). Although the UPR was originally identified as a conserved signaling pathway, functioning to maintain essential ER homeostasis, numerous studies indicate that the UPR has broader functions and plays an essential role in maintaining hepatic lipid homeostasis. Recent studies have shown that the pharmacologic ER stress inducers increase *de novo* lipogenesis and lipid droplet formation in hepatocytes by up-regulating a subset of genes encoding key lipogenic trans-activators and enzymes (Lee et al., 2012).

Hepatic lipid homeostasis is controlled by numerous transcription factors and nuclear receptors. The SREBPs are master regulators of lipid homeostasis (Eberle et al., 2004) and play a critical role in *de novo* lipid biosynthesis (Amemiya-Kudo et al., 2002). SREBP-1 controls fatty acid and triglyceride biosynthesis, while SREBP-2 controls cholesterol metabolism and LDL receptor expression. SREBPs, are basic-helix-loop-helix-leucine zipper (bHLHLZ) transcription factors bound to the ER membranes as an inactive precursor (Brown and Goldstein, 1997). The regulation of SREBP activity is controlled within the ER by the interaction of SCAP with insulin regulated proteins (Insigs; Yang et al., 2002). The Insigs can cause ER retention of the SREBP-SCAP complex and prevent the activation of SREBPs. When the sterol level is low, Insigs are disassociated with SCAP, which allows the SREBP-SCAP complex to migrate to the Golgi, where SREBPs are processed into active forms by S1P and S2P (Rawson, 2003; Lee and Ye, 2004). The activated SREBPs are subsequently translocated into nucleus, where SREBPs regulate the expression of various genes involved in lipid metabolism by binding to the sterol regulatory element of their target genes (Horton et al., 2002a,b; Radhakrishnan et al., 2007). It has been shown that ER stress induces proteolytic activation of SREBPs by increasing the turnover of Insig-1 (Lee and Ye, 2004). The expression of Insigs is regulated by insulin and FXRα (Yabe et al., 2003; Hubbert et al., 2007). Overexpression of hepatic Insigs has been shown to reduce hepatic lipogenesis (Engelking et al., 2004; Takaishi et al., 2004).

ER stress and lipid metabolism are tightly intertwined. The chronic ER stress is the most important contributor to metabolic diseases. Unresolved ER stress induces dysregulation of hepatic lipid metabolism (Fu et al., 2012). It is also well-documented that excess saturated fatty acids and cholesterol can induce ER stress and disrupt lipid metabolism in hepatocytes, macrophages and adipocytes (Fribley et al., 2009; Colgan et al., 2011; Anderson et al., 2012; Fu et al., 2012; Wang et al., 2013; Zha et al., 2013). The various components of the UPR signaling pathways play a role in the regulation of lipid metabolism.

### IRE1α-XBP1 PATHWAY

IRE1α-XBP1 pathway is one of three main branches of UPR, which has been identified as a critical regulator of hepatic lipid metabolism. Hepatic-specific deletion of IRE1α increased hepatic lipid levels and reduced plasma lipid by altering several genes involved in hepatic lipid metabolism under ER stress conditions such as C/EBPβ, C/EBPδ, peroxisome proliferator-activated receptor γ (PPARγ), and enzymes involved in triglyceride biosynthesis (Zhang et al., 2011). Although, these results suggest a plausible protective role of IRE1α from hepatic steatosis, the deletion of IRE1α blocks the basal level of the UPR in liver which may lead to an unresolved ER stress. Thus, it is still unclear whether the induction of lipogenesis genes in IRE1α^-^^/^^-^ mice is due to the loss of IRE1α function or elevated ER stress. Studies done by Lee et al. (2008) reported that disruption of hepatic XBP-1 significantly reduced serum triglyceride, cholesterol and fatty acids levels by decreasing *de novo* hepatic lipogenesis in mice. In addition, the IRE1α-XBP1 pathway is also involved in regulation of hepatic VLDL assembly and secretion (Wang et al., 2012). IRE1α is required for efficient secretion of VLDL and LDL from hepatocytes under the condition of ER stress (Zhang et al., 2011). A most recent study done by Rohrl et al. (2014) identified novel links between ER stress and hepatic cholesterol metabolism. Activation of acute ER stress reduced ABCA1 expression and induced ABCA1 redistribution to tubular perinuclear compartments in hepatocytes, which significantly diminished cholesterol efflux to apoA-I and HDL formation (Rohrl et al., 2014).

### PERK-ATF-4 PATHWAY

Protein kinase RNA-like ER kinase activation induces eIF2α phosphorylation, which causes translation attenuation that is required to protect against apoptosis in response to ER stress. Although the exact role of PERK in hepatic steatosis is still not completely understood, a recent report also suggests that antipsychotic drugs (APDs)-induced activation of PERK-p-eIF2α signaling pathway increases intracellular lipid accumulation through activation of SREBP-1c and SREBP-2 in hepatocytes (Lauressergues et al., 2012). Attenuation of eIF2α in GADD34 transgenic mice significantly altered the metabolism profile and reduced high fat diet-induced hepatic steatosis (Oyadomari et al., 2008). As a downstream transcriptional factor of the UPR, ATF4 avoids global suppression of protein expression induced by p-eIF2α due to a different upstream signaling pathway. It has been shown that ATF4 deficiency preferentially attenuated hepatic lipogenesis *via* down-regulation of PPARγ, SREBP-1c, ACC and SCD expression without affecting hepatic triglyceride production and fatty acid oxidation (Li et al., 2011; Xiao et al., 2013). A recent study further identified that activation of PERK-eIF2α-ATF4 pathway under ER stress condition is required for hepatic VLDL receptor up-regulation in hepatocytes, which is responsible for intracellular accumulation of triglycerides and hepatic steatosis (Jo et al., 2013). Furthermore, attenuation of global translation by activation of PERK-eIF2α pathway also decreases ApoB expression, which further promotes hepatic steatosis.

### ATF6 PATHWAY

Both ATF6 and SREBPs are activated by the same proteases (S1P and S2P) in the Golgi (Sakai et al., 1998; Ye et al., 2000; Horton et al., 2002b). Several independent studies have shown that ATF6 and XBP-1 share similar DNA binding specificities (Yoshida et al., 2000, 2001; Acosta-Alvear et al., 2007; Misiewicz et al., 2013). In addition, ATF6 and XBP-1 are able to form a heterodimer and regulate down-stream target genes (Yamamoto et al., 2007). Several recent studies in ATF6α knockout mice indicate that ATF6 also plays an important role in regulating hepatic lipid homeostasis (Rutkowski et al., 2008; Yamamoto et al., 2010). Similar to IRE1α, deletion of ATF6α does not result in an apparent phenotype under physiological conditions. However, under ER stress conditions, ATF6α knockout mice exhibited severe liver injury and hepatic steatosis caused by inhibition of fatty acid β-oxidation and VLDL formation (Yamamoto et al., 2010). In addition, the CHOP expression is significantly up-regulated while PPARα expression and ApoB-100 protein levels are decreased in the livers of ATF6α knockout mice (Rutkowski et al., 2008; Yamamoto et al., 2010). A recent study in zebrafish with fatty liver disease demonstrated that ER stress induces fatty liver disease. During chronic ER stress, ATF6 promotes steatosis. However, ATF6 prevents acute ER stress-induced steatosis. This study suggest that ATF6 can play both protective and pathological roles in fatty liver disease (Cinaroglu et al., 2011).

### ER STRESS MASTER REGULATOR-GRP78/BiP

GRP78/BiP is a glucose-regulated protein that functions as a molecular chaperone in the ER (Mote et al., 1998). As described in previous section, GRP78 acts as a master regulator of the activation of UPR signaling pathways. Numerous studies have indicated that ER stress is an important component of the hepatic steatosis and insulin resistance in obese rodent models (Kammoun et al., 2009; Chen et al., 2013; Teodoro-Morrison et al., 2013). GRP78 plays a critical role in maintaining hepatic lipid homeostasis. Overexpression of GRP78 prevents ER stress-induced SREBP-1c proteolytic cleavage and reduced hepatic steatosis (Kammoun et al., 2009). It also has been reported that overproduction of GRP78 prevents palmitate-induced ER stress and cytotoxicity in human HepG2 cells (Gu et al., 2010) and high fat diet-induced type 2 diabetes in mouse models (Teodoro-Morrison et al., 2013). A most recent study further indicated that GRP78 is able to prevent oxidative stress-induced injury by inhibiting lipid peroxidation (Suyama et al., 2014).

### C/EBP HOMOLOGOUS PROTEIN

C/EBP homologous protein is a proapoptotic transcriptional factor downstream of all three UPR signaling pathways. Numerous evidences suggest that CHOP activation promotes cell apoptosis and induces tissue injury. In absence of CHOP, both cells and animals are protected against various pharmacological and physiological insults (Tabas and Ron, 2011). It has been shown that CHOP deficiency attenuates cholestasis-induced liver fibrosis and methionine-choline-deficient (MCD) diet-induced steatohepatitis, fibrosis, and carcinogenesis in mice (Tamaki et al., 2008; Toriguchi et al., 2013). In a murine model of intragastric ethanol feeding, CHOP null mice have remarkable absence of hepatocellular apoptosis, but no protection against alcohol-induced steatosis (Ji et al., 2005). In human liver cell lines, saturated fatty acids induce ER stress and apoptosis *via* the PERK/ATF4/CHOP (Cao et al., 2012). Our recent studies suggest that CHOP is a major player in human immunodeficiency virus protease inhibitor-induced hepatic lipotoxicity in mice (Wang et al., 2013). Studies done by Chikka et al. (2013) further suggest CHOP has a non-apoptotic role in regulating hepatic metabolic genes during ER stress. In addition, a recent study reported that CHOP expression is up-regulated in human hepatocellular carcinoma (HCC) and two mouse HCC models. CHOP expression contributes to hepatic carcinogenesis by promoting inflammation and cell apoptosis (DeZwaan-McCabe et al., 2013). These studies indicate CHOP is a common contributing factor in ER stress-induced liver injury.

## THERAPEUTIC POTENTIAL TARGETING ER STRESS IN METABOLIC DISEASES

Chronic ER stress has been implicated in the pathogenesis of metabolic diseases such as a diabetes, obesity, cardiovascular diseases as well as fatty liver disease (Colgan et al., 2011; Malhi and Kaufman, 2011; Mollica et al., 2011; Bechmann et al., 2012; Cnop et al., 2012). Targeting the specific UPR signaling pathways to attenuate ER stress and UPR activation would provide opportunities in developing new therapeutic strategies in a wide array of diseases. Several studies have shown the promising effects of small chemical chaperones on alleviating the UPR activation in animal models, such as 4-phenylbutyric acid (4-PBA) and tauroursodeoxycholic acid (TUDCA; Heubi et al., 2002; Ozcan et al., 2006; Basseri et al., 2009; Engin and Hotamisligil, 2010; Lee et al., 2010). Both 4-PBA and TUDCA have been approved by US Food and Drug Administration (FDA) for treating children with urea-cycle disorders and cholestatic liver disease, respectively. However, more clinical studies are needed to validate the potential application of these chemical chaperones in treating ER stress-associated metabolic diseases.

## CONCLUSION

Hepatic lipid homeostasis requires integration of multiple signals. A significant amount of evidence indicates that activation of ER stress signaling pathways play a critical role in various diseases associated with dysregulation of hepatic lipid metabolism. Although acute ER stress response helps restore ER homeostasis, prolonged or chronic ER stress activation contributes to development of various metabolic diseases including NAFLD, type 2 diabetes, and atherosclerosis by inducing widespread pathologic apoptosis (Tabas and Ron, 2011). The balance of the UPR signaling pathways, such as ATF6, IRE1/XBP1, and PERK/ATF4, is critical for maintaining cellular homeostasis. However, the exact mechanisms underlying ER stress-induced disruption of hepatic lipid homeostasis remains to be fully identified. Recent studies have shown that ER stress is also closely linked to inflammation and autophagy, which are two important players in regulating hepatic lipid metabolism (Ogata et al., 2006; Yorimitsu et al., 2006; Hoyer-Hansen and Jaattela, 2007; Yorimitsu and Klionsky, 2007; Hotamisligil, 2010; Hummasti and Hotamisligil, 2010; Mollica et al., 2011; Qiu et al., 2011; Adolph et al., 2012; Hasnain et al., 2012; Kolattukudy and Niu, 2012). The contribution of autophagy to lipid metabolism has been reviewed in several excellent reviews (Singh, 2010; Amir and Czaja, 2011; Czaja, 2011; Ding et al., 2011; Lavallard et al., 2012). Elucidating the signaling pathways of ER stress and its intertwining with other intracellular signaling components not only furthers our current understanding of lipid metabolism in a central metabolic organ, but also helps develop an effective approach that can be used to treat patients with metabolic diseases.

A growing body of evidence links ER stress and UPR activation to diseases associated with lipid metabolism. The UPR signaling pathways and activation of transcription factors such as XBP1 and ATF6 have novel roles in controlling the transcriptional regulation of lipogenesis. While IRE1α itself is protective against ER-stress-induced lipogenesis and hepatic steatosis, its downstream mediator XBP1 promotes transcription of genes involved in fatty acid and cholesterol biosynthesis. Phosphorylation of eIF2α downstream of PERK affects the transcriptional activity of C/EBPs, PPARγ, and SREBP-1c thereby leading to lipid accumulation and hepatic steatosis under high-fat-diet conditions. Similar to IRE1α, ATF6α also protects against ER stress-induced steatosis and lipid droplet formation in mice. Furthermore, nuclear ATF6 attenuates SREBP2-mediated lipogenesis. The exact mechanisms by which ER stress signaling pathways affect lipid homeostasis are incompletely understood. Given the temporal differences in the activation of the three arms of the UPR, a closer examination of each branch of the UPR will allow for a better understanding of how various components of this signaling network impact lipogenesis and disease progression. Such studies will further enhance our understanding of the biological and pharmacological tools needed to effectively treat ER-associated diseases.

## Conflict of Interest Statement

The authors declare that the research was conducted in the absence of any commercial or financial relationships that could be construed as a potential conflict of interest.

## ABBREVIATIONS

ABCA1: ATP-binding cassette, sub-family A, member 1
ACC: acetyl-CoA carboxylase
ALD: alcoholic liver disease
ATF: activating transcription factor
C/EBP: CCAAT/enhancer-binding protein
CHOP: C/EBP homologous protein
DILD: drug-induced liver disease
eIF2α: eukaryotic translation initiation factor
ER: endoplasmic reticulum
FAS: fatty acid synthase
FXRα: farnesold X receptor α
GADD: growth arrest and DNA damage-inducible protein
HDL: high density lipoprotein
Insig: insulin-induced gene
IRE1: inositol requiring enzyme 1
JNK: c-Jun N-terminal kinase
LDL: low density lipoprotein
MCD: methionine-choline-deficient diet
NAFLD: non-alcoholic fatty liver disease
PERK: protein kinase RNA-like ER kinase
S1P: site 1 protease
S2P: site 2 protease
SCAP: SREBP cleavage activating protein
SCD: stearoyl-CoA desaturase
SREBP: sterol regulatory element binding protein
UPR: unfolded protein response
VLDL: very low density lipoprotein
VLDLR: VLDL receptor
XBP: X-box binding protein
